# Identification of a SmD3 epitope with a single symmetrical dimethylation of an arginine residue as a specific target of a subpopulation of anti-Sm antibodies

**DOI:** 10.1186/ar1455

**Published:** 2004-11-10

**Authors:** Michael Mahler, Marvin J Fritzler, Martin Blüthner

**Affiliations:** 1 Director Development and Production, Dr. Fooke Laboratorien GmbH, Neuss, Germany; 2 Professor of Medicine, Faculty of Medicine, University of Calgary, Calgary, Canada; 3 Vice Director of Autoimmune Diagnostics, Laboratory of Prof. Seelig and colleagues, Karlsruhe, Germany

**Keywords:** anti-Sm, autoantibody, ELISA, epitope, systemic lupus erythematosus

## Abstract

Anti-Sm antibodies, identified in 1966 by Tan and Kunkel, are highly specific serological markers for systemic lupus erythrematosus (SLE). Anti-Sm reactivity is found in 5–30% of SLE patients, depending on the autoantibody detection system and the racial background of the SLE population. The Sm autoantigen complex comprises at least nine different polypeptides. All of these core proteins can serve as targets of the anti-Sm B-cell response, but most frequently the B and D polypeptides are involved. Because the BB'Sm proteins share cross-reactive epitopes (PPPGMRPP) with U1 specific ribonucleoproteins, which are more frequently targeted by antibodies that are present in patients with mixed connective tissue disease, the SmD polypeptides are regarded as the Sm autoantigens that are most specific to SLE. It was recently shown that the polypeptides D1, D3 and BB' contain symmetrical dimethylarginine, which is a component of a major autoepitope within the carboxyl-terminus of SmD1. In one of those studies, a synthetic dimethylated peptide of SmD1 (amino acids 95–119) exhibited significantly increased immunoreactivity as compared with unmodified SmD1 peptide. Using immobilized peptides, we confirmed that the dimethylated arginine residues play an essential role in the formation of major SmD1 and SmD3 autoepitopes. Moreover, we demonstrated that one particular peptide of SmD3 represents a more sensitive and more reliable substrate for the detection of a subclass of anti-Sm antibodies. Twenty-eight out of 176 (15.9%) SLE patients but only one out of 449 (0.2%) control individuals tested positive for the anti-SmD3 peptide (SMP) antibodies in a new ELISA system. These data indicate that anti-SMP antibodies are exclusively present in sera from SLE patients. Thus, anti-SMP detection using ELISA represents a new serological marker with which to diagnose and discriminate between systemic autoimmune disorders.

## Introduction

Systemic rheumatic diseases are characterized by circulating autoantibodies to defined intracellular targets (for review [1]). Historically, among the earliest of these autoantibodies to be identified was anti-Sm, which was subsequently considered a serological hallmark of systemic lupus erythematosus (SLE) [2]. Thus, anti-Sm antibodies have been included among the American College of Rheumatology (ACR) criteria for classification of this disease [3]. In addition to autoantibodies that target the Sm complex, anti-double-stranded DNA (dsDNA), anti-proliferating cell nuclear antigen, anti-U1-RNP, anti-nucleosome, anti-histone, anti-Ro/SS-A, anti-La/SS-B, anti-ribosomal RNP, and anti-phospholipid antibodies are also frequently found in sera from SLE patients [1]. Of interest, certain SLE-associated autoantibodies have been shown to be present before the clinical onset of the disease and thus have high prognostic value [4].

On average, anti-Sm reactivity is found in 5–30% of patients with SLE, although the specific frequency depends on the detection system used and the racial and genetic makeup of the SLE population [5,6]. The Sm autoantigen is part of the spliceosomal complex that participates in the splicing of nuclear pre-mRNA [7]. The complex itself is comprised of at least nine different core polypeptides with molecular weights that range from 9 to 29.5 kDa [8]: B (B1; 28 kDa), B' (B2; 29 kDa), N (B3; 29.5 kDa), D1 (16 kDa), D2 (16.5 kDa), D3 (18 kDa), E (12 kDa), F (11 kDa) and G (9 kDa). All of these core proteins can be targets of the anti-Sm immune response, but the most prevalent response is to the B and D polypeptides, which are therefore considered the major antigens [8-10].

Because SmBB' share cross-reactive epitopes with U1-specific RNPs, which are more frequently targeted by antibodies that are present in patients with mixed connective tissue disease (MCTD), SmD is regarded as the Sm autoantigen that is most specific to SLE [11]. Within the SmD family, the SmD1/D3 reactivity pattern is at least four times more common than SmD1/D2/D3 recognition, with immunoreactivity to SmD1 being the most dominant [11]. Several linear and conformational epitopes have been mapped on the SmB and SmD proteins [12-14]. On SmD1 and SmBB' the major reactivity was found in the carboxyl-terminal regions [13-15]. The epitope PPPGMRPP, which occurs three times within the carboxyl-terminus of SmBB', was shown to crossreact with other proline-rich structures of spliceosomal autoantigens, including the U1-specific RNPs, and of retroviral proteins such as HIV-1 p24gag [16]. Follow-up studies and immunization experiments revealed this motif to be consistently the earliest detectable SmBB' epitope, indicating that it acts as a potential starting point for epitope-spreading events associated with the SmBB' molecule and SmD polypeptides [17,18].

A recent study [19] identified five linear epitopes on SmD2 and four on SmD3 that were distributed along the full length of the molecules. All of these epitopes share basic properties and are exposed on the surface of the protein, rendering them antigenic [19]. One of the B-cell epitopes on SmD3 (epitope 4; amino acids 104–126) exhibited sequence similarity with an antigenic region from the SmD1 protein, and this may account for some cross-reactivity [19]. For diagnostic purposes, a synthetic peptide corresponding to the carboxyl-terminal domain of SmD1 was used to develop an ELISA system with diagnostic sensitivities and specificities ranging from 36% to 70% and from 91.7% to 97.2%, respectively [6,20]. It was recently shown that the polypeptides D1, D3 and BB' contain symmetrical dimethylarginine (sDMA), which constitutes a major autoepitope within the carboxyl-terminus of SmD1 [21,22].

The aims of the present study were to develop a peptide-based ELISA system for the detection of anti-Sm antibodies and to evaluate the diagnostic properties of the peptide assay. Moreover, epitope-mapping experiments were performed to shed more light on the controversial findings of the importance of sDMA residues within the SmD1 and SmD3 sequences and their relationship to SLE.

## Methods

### Patients and sera evaluated

Sera (*n *= 628) were collected from patients suffering from SLE (*n *= 176), rheumatoid arthritis (*n *= 86), Sjögren's syndrome (*n *= 24), MCTD (*n *= 26), systemic sclerosis (*n *= 26) and polymyositis/dermatomyositis (*n *= 13), as well as from patients with overlap syndromes (*n *= 8). All patients were classified according to the ACR criteria for each disease [3,23-27]. Clinical, serological and demographic data were available from 101 SLE patients (SLE panel 1). This cohort contained 34 samples from white patients, 51 from black patients, five from hispanic patients, one from an east Indian patient, and one from an oriental patient. The racial background of four patients was not known. Of these patients, 13 were male and 86 female (in two the sex was unknown), and the mean age was 40 years (range 16–80 years). The sera were kindly provided by Drs R Mierau and E Genth (Rheumaklinik Aachen, Aachen, Germany), Prof. Dr MJ Fritzler (University of Calgary, Calgary, Canada) and by Labor Limbach (Heidelberg, Germany).

To assess further the assay specificity, we analyzed a group of sera from patients with infectious diseases (*n *= 77), including hepatitis C virus (*n *= 30), cytomegalovirus (*n *= 22) and Epstein–Barr virus (EBV; *n *= 25) infections, as well as from 192 healthy blood donors. All sera were stored at -80°C until use. For the epitope-mapping study, a panel of five sera (all from SLE patients) containing anti-Sm antibodies that were available in greater quantities preselected by ELISA (Varelisa^® ^Sm; Pharmacia Diagnostics, Freiburg, Germany) was used. Autoimmune sera with antibody specificities other than anti-Sm were selected as negative controls, including anti-RNP, anti-SS-A (Ro), anti-SS-B (La), anti-PM/Scl, anti-centromere protein (CENP) and anti-Scl-70.

### Serological characterization of randomly selected systemic lupus erythematosus sera

The sera from SLE patients represented in panel 1 with clinical, serological and demographic data, as well as the autoimmune controls, were tested for autoantibodies to histones (cutoff 30 U/ml), dsDNA (cutoff 55 U/ml) and the Sm complex (cutoff 15 U/ml, or ratio 1) using quantitative ELISA tests (Varelisa^®^; Pharmacia Diagnostics; catalog nos 14196, 16296 and 16496). SLE sera and samples that exhibited unexpected results were also measured using the semiquantitative Split anti-nuclear antibody (ANA) profile (Pharmacia Diagnostics; cutoff ratio 1). The latter assay contains the autoantigens U1-68 kDa, U1-A, U1-C, SmBB', SmD, Ro-52, Ro-60 and La. All ELISAs were performed in accordance with the manufacturer's instructions.

### Reference serum panels

The US Centers for Disease Control and Prevention (CDC) ANA serum panel [28] and the Association of Medical Laboratory Immunologists (AMLI) samples [29] were used to characterize the new Sm peptide-based immunoassay.

### Epitope-mapping with immobilized oligopeptides

The published sequences of SmD1 (P13641) and SmD3 (P43331) were used to synthesize overlapping 15 mer peptides with a pipetting robot (ASP222; Abimed, Langenfield, Germany), in accordance with the protocol described by Gausepohl and Behn [30-33]. The carboxyl-terminal extensions of both polypeptides were synthesized with an offset of two amino acids (13 amino acid overlap). Each arginine-containing peptide was synthesized as three variants, one with natural arginine, one with sDMA and one with asymmetrical dimethylarginine at the respective positions. In addition, a highly reactive SmD3 peptide was synthesized with certain combinations of natural arginine and sDMA. Following completion of the peptide synthesis, nonspecific binding sites were blocked by overnight incubation of the membranes in blocking buffer (2% milk in Tris-buffered saline) at room temperature. After one washing step for 5 min, the membranes were incubated with serum samples diluted 1:100 in blocking buffer for 2 hours at room temperature. Unbound antibodies were removed by three washing steps in Tris-buffered saline–0.2% Tween. The membranes were incubated for 75 min at room temperature in a peroxidase-conjugated goat-anti-human IgG antibody (Dianova, Hamburg, Germany) that was diluted 1:5000 in blocking buffer. Unbound secondary antibodies were removed by three changes of Tris-buffered saline. Finally, bound antibodies were visualized using the ECL detection system (Amersham Bioscience, Freiburg, Germany).

### Synthesis of the SmD3 peptide

The candidate SmD3 peptide (SMP) identified in the epitope-mapping study (^108^AARG *sDMA *GRGMGRGNIF^122^) was synthesized with an additional cysteine residue at the carboxyl-terminus, in accordance with Fmoc-chemistry at the Peptide Specialty Laboratories GmbH (Heidelberg, Germany). sDMA (Ref. B-3345.0001) was purchased from Bachem AG (Bubendorf, Switzerland) and used for synthesis. Crude fraction was purified using high-performance liquid chromatography. Quality and purity of the peptide was assessed by mass spectrometry and analytical high-performance liquid chromatography. The molecular mass was found to be 1708.1 Da (average; monoisotopic mass 1706.9), and a purity in excess of 95% was identified.

### SmD3 peptide ELISA

Microtiterplates (Maxisorb, Nunc, Denmark) were coated with the uncoupled 16 mer peptide at a concentration of 2.5 μg/ml in phosphate-buffered saline (pH = 7.6). After an incubation time of 15 hours at 15°C, the plates were blocked with 1% bovine serum albumin in phosphate-buffered saline for 30 min at room temperature. The assay was performed in accordance with the general protocol for the Varelisa^® ^system (Pharmacia Diagnostics). In brief, following a prewashing (300 μl/well) step, the serum samples were diluted 1:101 in sample buffer (phosphate-buffered saline, containing bovine serum albumin and Tween), added to the wells and then incubated for 30 min (100 μl/well). After three washing steps (300 μl/well), horseradish peroxidase conjugated anti-human IgG was added and incubated for 30 min (100 μl/well). Visualization was done by incubation in 3,3',5,5'-tetra-methyl benzidine substrate for 10 min (100 μl/well), and the reaction was terminated by adding 50 μl stop solution (0.5 mol/l H_2_SO_4_) to each well. All steps were carried out at room temperature.

A standard curve was developed using a highly reactive index serum that bound the SmD3 peptide and had a defined reactivity of 40,000 U/ml. The curve was plotted at six standard points (0, 3, 7, 16, 40 and 100 U/ml). Each serum sample was tested in duplicate and serum samples that had reactivity above the assay range were serially diluted to 1:500, 1:2500, 1:12,500 and 1:62,500.

To define further the assay characteristics, 192 normal human sera were assayed in accordance with the instructions for use. Blood donors exhibited a reactivity range of 0.4–11.5 U/ml, a mean value of 2.2 U/ml and a standard deviation of 1.2 U/ml. The cutoff was set at 13 U/ml following receiver operating characteristic (ROC) analysis. Positive predictive values (PPVs) and negative predictive values (NPVs) were calculated at different cutoff values using Analyse-it software (Version 1.62; Analyse-it Software Ltd, Leeds, UK).

### Precision and reproducibility

Measurements of imprecision (interassay and intra-assay variability) were taken over four and six replicates, respectively. To assess the precision of the anti-SMP ELISA, suitable anti-Sm sera – a low value sample (L), a medium value sample (M) and a high value sample (H) – were assayed in five independent tests on one day (interassay) or in a single run (intra-assay). For within-run precision, the L, M and H samples were measured in six replicates on one solid phase. The precision data were calculated using analysis of variance.

### Linearity

Linearity was analyzed by testing dilutions (1:1, 2:3, 1:2, 1:4, 1:8, 1:16, 1:32) of the highest standard point (S6) and of the high value sample from the precision analysis (H). For each dilution point, a ratio of the measured reactivity to the expected value was calculated, and 1 was subtracted from this quotient.

## Results

### Epitope fine mapping of the carboxyl-terminal extensions of SmD1 and SmD3

**Figure 1:**
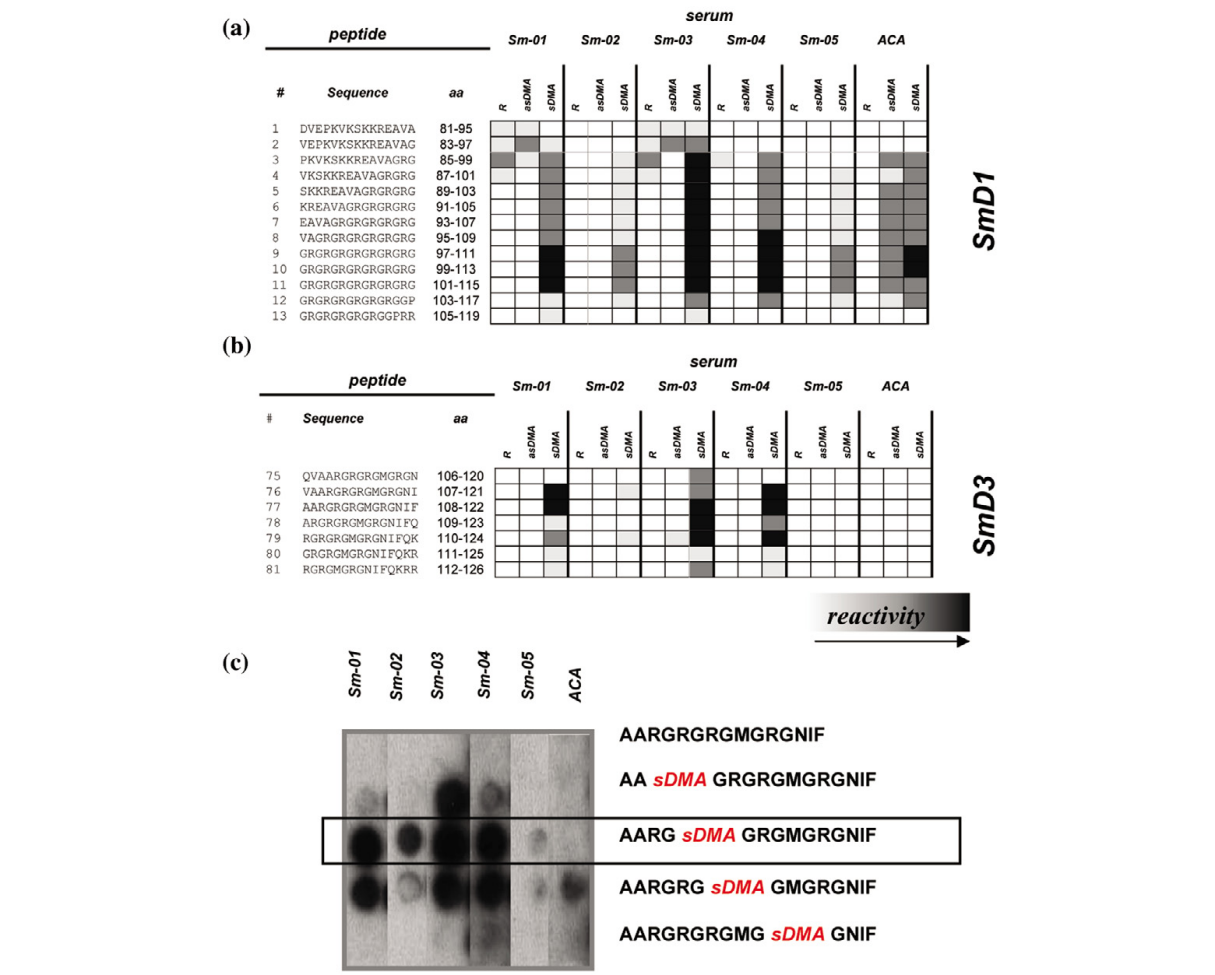
Epitope analysis of SmD1 and SmD3. Carboxyl-terminal regions of **(a) **SmD1 and **(b) **SmD3 were synthesized as peptide arrays (15 mers; two amino acids offset) and probed with patient sera. Each arginine containing peptide was synthesized as three variants, one with natural arginine (R), one with symmetrical dimethylarginine (sDMA) and one with asymmetrical dimethylarginine (asDMA) at the respective positions. In addition, a highly reactive SmD3 peptide was synthesized with certain combinations of natural arginine and sDMA. A significant effect of dimethylation of arginine residues on the antigenicity of SmD derived peptides was observed (black squares indicate strong reactivity; white indicate no reactivity). Binding of an anti-Sm negative serum sample (Varelisa^® ^Sm) that contained anti-centromere antibodies (ACA) could be observed with SmD1 but not with SmD3 peptides. Thus, the immunoreactive peptide no. 77 was further tested in **(c) **a replacement experiment. The SmD3 peptide exhibited exclusive reactivity with the Sm-positive sera.

To evaluate the effect of arginine dimethylation on the antigenicity of SmD1 and SmD3 and to localize relevant epitopes on both polypeptides, a panel of anti-Sm sera was tested for reactivity with peptide arrays (15 mer, two offset) covering the carboxyl-terminal region of SmD1 (P13641) and SmD3 (P43331). The results show that dimethylation of arginine residues significantly affects the binding of anti-Sm antibodies to carboxyl-terminal SmD1 and SmD3 peptides (Fig. 1). All anti-Sm sera exhibited increased binding to SmD1 peptides containing sDMA as compared with those containing unmethylated arginine (Fig. 1a). In particular, peptides that exclusively consist of glycine and sDMA repeats exhibited strong reactivity with the antibodies (peptide nos 9, 10 and 11). Nevertheless, SmD1 peptides containing sDMA represent a rather unspecific substrate for anti-Sm antibodies because they were also bound by sera that contained anti-centromere antibodies. Interestingly, those anti-centromere antibodies also bound to peptides containing the asymmetrical form of DMA but to a lesser extent.

Binding experiments with peptides derived from SmD3 yielded similar results. Only SmD3 peptides containing sDMA reacted with anti-Sm antibodies, confirming the importance of the symmetrical dimethylation of arginine residues (Fig. 1b). In contrast to SmD1, none of the control sera reacted with SmD3 derived peptides, which reflects high binding specificity. One particular peptide (no. 77; ^108^AA *sDMA *G *sDMA *G *sDMA *GMG *sDMA *GNIF^122^) was strongly recognized by three out of five anti-Sm sera. Using a mutational analysis in which arginine residues of peptide no. 77 (^108^AARGRGRGMGRGNIF^122^) were successively replaced by sDMA, we were able to show that a particular peptide with a single dimethylated arginine residue at position 112 exhibited immunoreactivity with all of the five anti-Sm sera but not with the control sera (Fig. 1c). Thus, by introducing only one sDMA and at a defined position (amino acid 112) in SmD3, the sensitivity of binding to this peptide (^108^AARG *sDMA *GRGMGRGNIF^122^; SMP) was remarkably increased without loss in specificity. This candidate peptide was subsequently synthesized as a soluble antigen and used as a substrate in ELISA.

### Immunoserological characterization of the systemic lupus erythematosus patient cohort

In order to characterize the SLE cohort with regard to autoantibody profiles, 101 SLE patient sera were tested for U1-68 kD, U1-A, U1-C, SmBB', SmD, Ro-52/SS-A, Ro-60/SS-A, La/SS-B, histone, dsDNA and β_2_-glycoprotein I reactivity. The prevalences of the different autoantibodies were as follows: 15.8% for U1-68, 24.8% for U1-A, 25.7% for U1-C, 21.8% for SmBB', 15.8% for SmD, 21.8% for Ro-52, 47.5% for Ro-60, 21.8% for La, 37.6% for histone, 51% for dsDNA and 17% for β_2_-glycoprotein I. The prevalences were therefore consistent with those in previous studies [1,6]. Thus, with regard to their autoantibody profiles, our SLE cohort appears to be representative of SLE patients in general.

### Anti-SmD3 peptide ELISA

A 15 amino acid soluble peptide exhibited highest sensitivity and specificity in the SPOT assay (^108^AARG *sDMA *GRGMGRGNIF^122^) was, for coupling purposes, synthesized with an additional cysteine at the carboxyl-terminus. Nevertheless, the uncoupled 16 mer peptide was subsequently used to develop an ELISA system based on the general protocol of the Varelisa^® ^tests (Pharmacia Diagnostics).

### Assay performance characteristics

**Figure 2:**
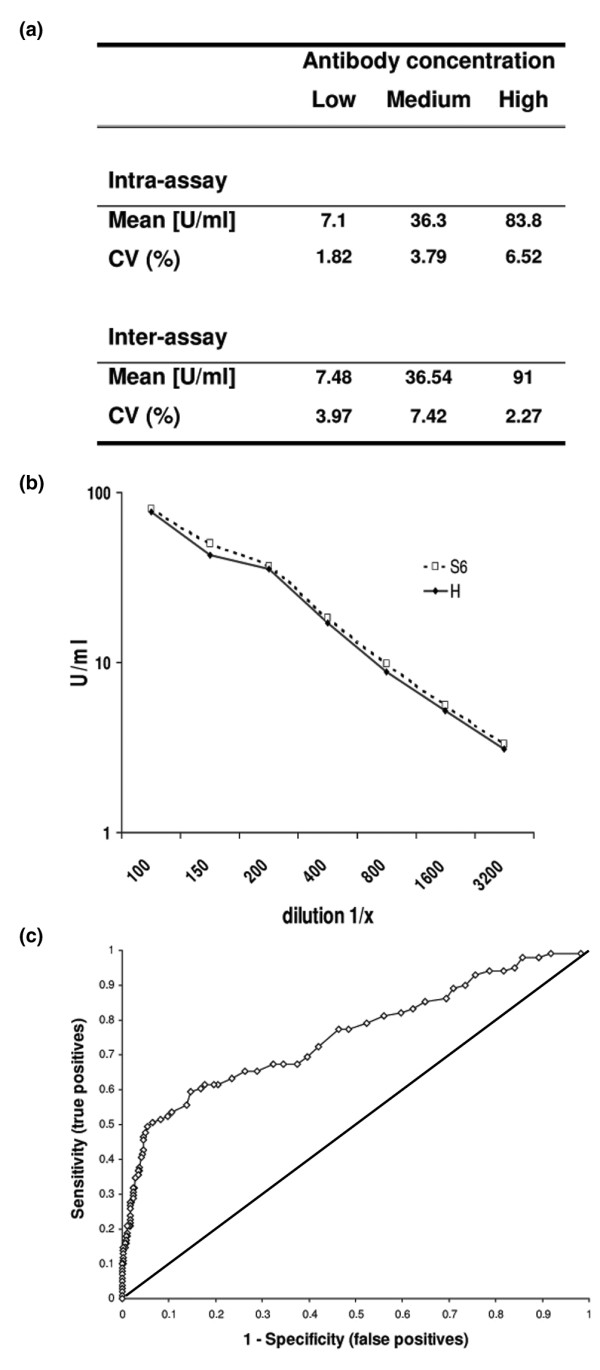
Assay performance characteristics of the anti-SmD3 peptide (SMP) assay. **(a) **Intra-assay and interassay variability, **(b) **linearity, and **(c) **receiver operating characteristic analysis. The intra-assay and interassay variability, expressed as coefficient of variation in percentage (CV%), of three samples ranged from 1.82 to 6.52% and from 2.27 to 7.42%, respectively. Serial dilution series of two samples with high titres of anti-Sm antibodies (S6 and H) exhibited a linear binding response (<20% deviation). Definition of the cutoff, using receiver operating characteristic (ROC) analysis, was performed with SLE and control sera.

To evaluate the performance of the assay, the precision, reproducibility and linearity were analyzed. The intra-assay and interassay variabilities (coefficient of variation in %) for three samples ranged from 1.82% to 6.52% and from 2.27% to 7.42%, respectively. Even after five serial dilutions, two samples exhibited a linear range of reactivity (<20% deviation). The cutoff was defined by ROC analysis, performed with SLE and control sera. The assay performance characteristics of the new anti-SMP test are summarized in Fig. 2, including intra-assay and interassay variability (Fig. 2a), linearity (Fig. 2b), and ROC analysis, PPV, NPV and efficiency (Fig. 2c).

To determine the cutoff, the focus was set to yield a high specificity and a technical cutoff was defined at 13 U/ml. Sera from 176 SLE patients, 181 other autoimmune patients, 77 patients with infectious diseases, and from 192 normal donors were analyzed in the new ELISA system (Table 1). Twenty-eight SLE patients (15.9%) tested positive for anti-SMP antibodies, exhibiting a significantly increased reactivity of up to 1190 U/ml with a mean value of 43.0 U/ml (standard deviation 160.2 U/ml). Sera from patients with related disorders had significantly reduced reactivity (mean 3.36 U/ml). Only one patient in the rheumatoid arthritis group had a positive test result (24.6 U/ml). None of the remaining control individuals, including patients suffering from systemic sclerosis (*n *= 26), polymyositis/dermatomyositis (*n *= 13), MCTD (*n *= 26), Sjögren's syndrome (*n *= 24), or infectious diseases (*n *= 77), exhibited reactivity to the SmD3 peptide. The serum samples from patients with infectious diseases demonstrated reduced reactivity (mean 0.67 U/ml; top value 3.3 U/ml), even when compared with sera from healthy donors (mean 2.21 U/ml; top value 11.5 U/ml). The highest assay value in the infectious disease sera was found in patients with EBV infection (3.3 U/ml).

In summary, 28 samples in the SLE cohort (*n *= 176) and only one serum sample from the control group (*n *= 449; 0.2%) tested positive. This resulted in a diagnostic specificity of 99.8% and a sensitivity of 15.9%. PPV, NPV and diagnostic efficiency were calculated at 96.6%, 75.3% and 76.3%, respectively (Fig. 2c). These data indicate that, within the assay parameters used here, anti-SMP antibodies appear to be exclusively present in sera from SLE patients. Apart from anti-SMP reactivity, it is interesting to note that the positive rheumatoid arthritis serum contained high titres of antibodies to U1-RNPs 68 kDa (ratio 4.5), U1-C (ratio 9.4) and histone (133.8 U/ml). Anti-SmBB'and anti-SmD titres, as determined by ELISA, were elevated when compared with controls, but they were still below the cutoff values. No reactivity could be found to U1-A, Ro-52, Ro-60, La, dsDNA, or β_2_-glycoprotein.

### Correlation with other autoantibodies

A statistical evaluation was performed using sera from a cohort of 101 patients with clinically defined SLE to evaluate correlations between anti-SmD3 peptide antibodies and other autoantibodies. Significant correlations were found with dsDNA (*P *= 0.0058, χ^2 ^= 7.6), U1-68 (*P *< 0.0001, χ^2 ^= 15.42), U1-A (*P *< 0.0001, χ^2 ^= 25.49), U1-C (*P *< 0.0001, χ^2 ^= 18.05), SmBB' (*P *< 0.0001, χ^2 ^= 24.04) and SmD (*P *< 0.0001, χ^2 ^= 38.76), but not to histone (*P *= 0.0259, χ^2 ^= 4.96), La (*P *= 0.8747, χ^2 ^= 0.02), Ro-52 (*P *= 0.4034, χ^2 ^= 0.7), Ro-60 (*P *= 0.0143, χ^2 ^= 6.0) and β_2_-glycoprotein antibodies (*P *= 0.3819, χ^2 ^= 0.74; Table 2). When reactivity with components of the Sm complex was evaluated, five samples of the clinically defined SLE patients (*n *= 101) reacted with the purified SmD antigen, but not with SMP. The remaining 11 SmD positive sera (68.8%) also tested positive in the new anti-SMP ELISA. Interestingly, among the patients studied we found four (nos 89, 92, 20627 and 9811) who fulfilled SLE criteria and were all anti-SmD negative, but exhibited anti-SMP reactivities of 15.4, 21.3, 41.3 and 13.9 units, respectively.

### Correlation with racial and clinical parameters

When correlating autoantibody specificities with race, there was a statistically significant association of autoantibodies to U1-68 kDa (*P *= 0.002), U1-A (*P *< 0.0001), U1-C (*P *= 0.0002), SmBB' (*P *= 0.0004), dsDNA (*P *= 0.0128) and SmD (*P *= 0.0002) with black race among SLE patients. There was no statistically significant association of other autoantibody specificities, including SmD3 peptide (*P *= 0.0253), Ro-52 (*P *= 0.8023), Ro-60 (*P *= 0.0399), La (*P *= 0.7137) and histones (*P *= 0.9831), with the race of the patients under investigation (data not shown). In addition, there was no significant correlation of SMP reactivity with renal (*P *= 0.2810) or central nerve system involvement (*P *= 0.5066).

### Reference panels

The CDC and the AMLI reference panels for ANA were evaluated using the new SMP ELISA. Increased titres were found in ANA 1 (10.3 U/ml) and ANA 5 (910 U/ml) from the CDC panel and in samples I (1420 U/ml) and J (13.9 U/ml) from the AMLI panel [28,29] (Table 3).

## Discussion

In the present study we analyzed the anti-Sm immune response directed toward the Sm antigens D1 and D3, which are considered SLE-specific autoantigens [11]. Using immobilized peptides prepared by the SPOT technology, it was shown that symmetric dimethylation of arginine residues plays an important role in the B-cell epitope recognition of both autoantigens. This observation is in accordance with the findings of Brahms and coworkers [21] but it is not in keeping with those of Riemekasten and colleagues [20]. In addition, we found the specificity of antibody binding to SmD3 peptides to be higher than that to SmD1 peptides, both of which were prepared using the SPOT method.

McClain and colleagues [19] described four antigenic regions on SmD3, of which antigenic region 4 encompasses amino acids 104–126. In their study, peptides synthesized on pins were subjected to analysis without using the modified form of arginine. In our study, we found reactivity within this region only when natural arginine was replaced by sDMA. These apparently contradictory results might be explained by the use of different sera or methology, and/or by the different peptide length used in the two studies. Three out of five of our sera specifically recognized the peptide ^108^AA *sDMA *G *sDMA *G *sDMA *GMG *sDMA *GNIF^122^. Interestingly, the dimethylation of only one arginine at a defined position (amino acid 112) was able to increase further the sensitivity of this particular peptide without loss of specificity.

Although it has been shown that all arginine residues within the peptide ^108^AA *sDMA *G *sDMA *G *sDMA *GMG *sDMA *GNIF^122 ^become dimethylated *in vivo*, it is unclear whether the identified peptide with single dimethylation occurs *in vivo *and thus serves as the triggering epitope, or rather whether it represents an artificial structure that is more suitable for *in vitro *assays [21].

Fewer than 20 proteins have been identified during the past 40 years as containing dimethylated arginines [34]. The two major catalyzing enzymes of this reaction are the type I and type II protein arginine methyltransferases, which preferentially methylate arginines located in RG clusters. Recently, however, using arginine methyl-specific antibodies and HeLa cell extracts, it was shown that more than 200 proteins contained symmetrically dimethylated arginines; among these were a remarkable number of known autoantigens [34]. Further studies are necessary to screen known autoantigens containing dimethylated arginine residues for epitopes.

Based on data from epitope analysis, we used a candidate peptide (^108^AARG *sDMA *GRGMGRGNIF^122^) to develop an ELISA system. The new anti-Sm assay (anti-SMP) had a sensitivity of 15.9% and a specificity of 99.8% for SLE, resulting in a high PPV (96.6%) and NPV (75.3%), and a remarkable diagnostic efficiency of 76.3%. Therefore, this test appears to offer a new approach to serological evaluation and diagnosis of SLE.

Because no international 'gold standard' is available for detection of anti-Sm antibodies, we compared results with the new peptide-based ELISA with the results of an anti-Sm ELISA using purified Sm antigen (Varelisa^® ^Sm; Pharmacia Diagnostics). Using this approach, we found comparable sensitivities but significant differences in the specificity. The specificity of the conventional ELISA (88%) was significantly lower than the specificity of the new peptide-based assay (99.8%; data not shown). Further studies are in progress to compare the assay performance of the anti-SMP assay with that of other commercially available anti-Sm immunoassays. For epitope-mapping, anti-Sm sera from SLE patients were preselected, based on ELISA results (Varelisa^® ^Sm). That this selection method might have affected the results of epitope-mapping cannot be excluded. Nevertheless, the high sensitivity and specificity of the SmD3 peptide with a single dimethylated arginine could be confirmed using the soluble peptide in ELISA.

Evaluation of the biochemical properties of the identified Sm epitopes suggested that the isoelectric point (pI) of the peptide can be regarded as a predictor of antigenicity on the Sm complex. On U1-RNP-A, SmB' and SmD1, the average pI of antigenic regions was 10.4 (nonantigenic 6.0) and on SmD2 and SmD3 the pIs were 9.0 or higher [19]. These findings fit well with the observed pI (>12.88) of the SMP. Further investigation is warranted to determine whether the basic character of the epitope simply increases the probability of surface exposure of these regions and thus accessibility for immune recognition.

### Epstein–Barr virus, Epstein–Barr virus nuclear antigen 1 and anti-SmD antibodies

Epitope-mapping studies on SmD1 have identified an epitope motif (amino acids 95–119) that crossreacts with a homologous sequence (amino acids 35-58) of the EBV nuclear antigen 1 [35,36]. A more recent study showed that this epitope also crossreacts with a homologous region of SmD3 containing glycine and arginine repeats (RGRGRGMGR) [19]. It is also evident that GPRR (amino acids 114–119 on SmD1) represents a common crossreactive autoepitope motif, which is present not only on EBV nuclear antigen 1, but also on a variety of autoantigens including CENP-A, CENP-B, CENP-C, SmBB', SmD1 and Ro-52, to name but a few [30]. Thus, patients suffering from infectious mononucleosis or SLE-related disorders may have a positive carboxyl-terminal SmD1 or SmD3 ELISA that might be regarded as a false-positive result. Of interest, several studies have suggested an influence of EBV on the development of SLE-like conditions [37,38]. Among the 25 EBV disease controls we evaluated, we found no false-positive samples, confirming the suggested high specificity of the anti-SMP assay. Therefore, we consider the use of EBV-positive sera with high titres of EBV-associated antibodies to be important reagents for developing highly specific and reliable anti-SmD immunoassays. Unfortunately, other investigators did not include an EBV patient group in the evaluation of anti-Sm antibody assays [20].

### Correlations with other autoantibodies

Coincident reactivity with dsDNA and Sm antigens has been reported by several authors [39-41]. Although in those studies full-length SmD was used, in our investigation there was also a correlation of anti-dsDNA and anti-SMP reactivity (*P *= 0.0058, χ^2 ^= 7.6). Apart from DNA we found also a positive correlation of anti-SMP antibodies with U1-68 (*P *< 0.0001, χ^2 ^= 15.42), U1-A (*P *< 0.0001, χ^2 ^= 25.49), U1-C (*P *< 0.0001, χ^2 ^= 18.05), SmBB' (*P *< 0.0001, χ^2 ^= 24.04) and SmD (*P *< 0.0001, χ^2 ^= 38.76), but not to histone (*P *= 0.0259, χ^2 ^= 4.96), La (*P *= 0.8747, χ^2 ^= 0.02), Ro-52 (*P *= 0.4034, χ^2 ^= 0.7), Ro-60 (*P *= 0.0143, χ^2 ^= 6.0) and β_2_-glycoprotein (*P *= 0.3819, χ^2 ^= 0.74) antibodies. Whether the observed associations are caused by cross-reactivity or by different autoantibody species that often occur simultaneously remains unclear. Preliminary results of inhibition experiments have shown no inhibiting effect of the SmD3 peptide on the binding of anti-Sm antibodies to the native Sm antigen in ELISA (Varelisa^® ^Sm; data not shown). The absence of inhibition can be explained by the variety of different anti-Sm antibody subpopulations and by the variety of corresponding epitopes. Recently, two polyclonal antibodies (SYM10, SYM11) were generated that specifically bind to the symmetrical form of dimethylarginine and react with a variety of other known autoantigens [42]. Those antibodies may shed more light on the correlation of anti-SMP antibodies with other known autoantibody specificities.

### Reference sera

The reference sera for ANA obtained from the CDC and the AMLI were tested using the anti-SMP assay [28,29]. Although sample J (AMLI) was defined as a RNP-positive serum, we found reactivity to the SMP (13.9 U/ml). None of five investigators found precipitating anti-Sm antibodies, and only two out of 21 reported Sm reactivity in their enzyme immunoassay in this serum, which was derived from a patient with SLE. In the immunoblot of the AMLI study, both serum I and serum J exhibited reactivity to the SmD proteins. Surprisingly, the reactivity to SmD3 was significantly greater in sample J than in serum I. Thus, the anti-SMP antibody test had a higher sensitivity and clinical accuracy than the anti-Sm tests used in most of the participating laboratories [29].

The apparent disparity between the results of the present study and those of Riemekasten [20] and Brahms [21] and their groups might be explained by the existence of different epitopes on the carboxyl-terminal extensions of SmD1 and SmD3. The peptide (amino acids 83–119) [20] may form a conformational epitope, whereas the shorter peptides used in the second study contain primarily linear, sDMA-dependent binding sites [21]. Furthermore, the reduced reactivity against the full-length SmD1 [20], as compared with SmD1_83–119 _peptide, suggests that this epitope represents a cryptic structure. This observation raises the issue of which epitopes are 'seen' *in vivo *and which ones play a central role in the pathogenesis of SLE. In a recent study [43] it was observed that the injection of SmD1_83–119 _fused to a carrier protein is able to accelerate the pathogenic process in SLE-prone mice.

### Summary

In the present study we showed that dimethylation of arginine residues of the major SmD1 and SmD3 autoepitopes results in remarkably increased binding by SLE autoantibodies. Moreover, it could be shown that one particular SmD3 peptide represents a highly specific substrate for detecting a subclass of anti-Sm antibodies by ELISA. At a defined cutoff value of 13 U/ml, the sensitivity was 15.9% and the specificity was 99.8%, yielding a diagnostic efficiency of 76.3%.

## Conclusion

Based on the findings of the present study, we conclude that anti-SMP antibodies are exclusively present in sera from SLE patients and that the new anti-SMP ELISA test appears to offer a new serological reagent that will improve our ability to diagnosis SLE and to discriminate SLE from other autoimmune and infectious diseases.

## Abbreviations

ACR = American College of Rheumatology; AMLI = Association of Medical Laboratory Immunologists; ANA = anti-nuclear antibody; CDC = Centers for Disease Control and Prevention; CENP = centromere protein; dsDNA = double-stranded DNA; EBV = Epstein–Barr virus; ELISA = enzyme-linked immunosorbent assay; MCTD = mixed connective tissue disease; NPV = negative predictive value; pI = isoelectric point; PPV = positive predictive value; ROC = receiver operating characteristic; sDMA = symmetrical dimethylarginine; SLE = systemic lupus erythematosus; SMP = SmD3 peptide.

## Competing interests

MM receives royalties for the commercial ELISA system from Pharmacia Diagnostics (Freiburg, Germany).

## Authors' contributions

MM planned and initiated the present study. He carried out the epitope-mapping of SmD1 and SmD3, and developed and evaluated the ELISA system. Based on the results he filed a draft verison of the manuscript. MB advised MM regarding the planning of the epitope -mapping experiments and contributed to the preparation of the manuscript. MF delivered clinically defined sera, advised MM on evaluating the clincal part of the study, and contributed to the preparation of the manuscript.

## Figures and Tables

**Table 1:** Results of ELISA using SmD3 peptide with systemic lupus erythematosus and various control sera

Sera (*n*)	Number (%) of anti-SMP positive sera	Mean value (U/ml)	Top value (U/ml)
SLE (176)	28 (15.9)	43.0	1190.0
Rheumatic diseases (181)	1 (0.6)	2.2	24.6
RA (86)	1 (1.2)	1.6	24.6
pSS (24)	0	1.9	3.9
MCTD (26)	0	3.1	12.8
SSc (26)	0	2.4	4.3
PM/DM (13)	0	2.8	9.6
Overlap syndromes (8)	0	2.1	3.6
Infectious diseases (77)	0	0.67	3.3
HCV (30)	0	0.42	1.1
CMV (22)	0	0.8	3.2
EBV (25)	0	0.78	3.3
Healthy individuals (192)	0	2.21	11.5

CMV, cytomegalovirus; EBV, Epstein – Barr virus; HCV, hepatitis C virus; MCTD, mixed connective tissue disease; PM/DM, polymyositis/dermatomyositis; pSS, primary Sjögren's syndrome; RA, rheumatoid arthritis; SLE, systemic lupus erythematosus; SMP, SmD3 peptide; SSc, systemic sclerosis.

**Table 2:** Association between anti-SmD3 peptide positivity and other autoantibody species in systemic lupus erythematosus

	Autoantibody to										
	
	U1-68 kD	U1-A	U1-C	SmBB'	SmD	Ro-52	Ro-60	La	histone	dsDNA	β_2_-Glycoprotein
SMP positive	8/16	12/25	11/26	11/22	11/16	5/22	12/48	4/22	10/38	13/51	4/16
Percentage	50%	48%	42.3%	50%	68.8%	22.7%	25%	18.2%	26.3%	25.5%	25%
*P*	<0.0001	<0.0001	<0.0001	<0.0001	<0.0001	0.4034	0.0143	0.8747	0.0259	0.0058	0.3891
χ^2^statistic	15.42*	25.49*	18.05*	24.04*	38.76*	0.7	6.0	0.02	4.96	7.6*	0.74

* χ^2 ^test: statistically significant (χ^2 ^> 7).

**Table 3:** Results of the reference sera from the CDC and AMLI in the new anti-SmD3 peptide ELISA

Sample ID	U/ml	Sample ID	U/ml	Diagnosis
ANA 1	10.3	SLR Research A	2.5	CREST
ANA 2	1.4	SLR Research B	2.9	Scl
ANA 3	1.9	SLR Research D	2.8	MCTD
ANA 4	2.5	SLR Research E	3.1	SS, SLE
ANA 5	910.0	SLR Research F	1.5	PM
ANA 6	3.1	SLR Research G	4.5	SS
ANA 7	0.0	SLR Research I	1420.0	SLE
ANA 8	0.1	SLR Research J	13.9	SLE
ANA 9	1.7	SLR Research K	0.0	HD
ANA 10	0.5	SLR Research L	0.2	HD

AMLI, Association of Medical Laboratory Immunologists; CDC, Center for Disease Control and Prevention; CREST, calcinosis cutis, Raynaud's phenomenon, esophageal dysfunction, sclerodactyly and telangiectasia; HD, healthy donor; MCTD, mixed connective tissue disease; PM, polymyositis; Scl, scleroderma; SLE, systemic lupus erythematosus; SS, Sjögren's syndrome.
